# Heterodimerization of T cell engaging bispecific antibodies to enhance specificity against pancreatic ductal adenocarcinoma

**DOI:** 10.1186/s13045-024-01538-5

**Published:** 2024-04-23

**Authors:** Alan W Long, Hong Xu, Brian H Santich, Hongfen Guo, Sayed Shahabuddin Hoseini, Elisa de Stanchina, Nai-Kong V Cheung

**Affiliations:** 1 Department of Pediatrics, Memorial Sloan Kettering Cancer Center, 1275 York Ave, New York, NY 10065 USA; 2 Department of pathology, Westchester Medical Center, Valhalla, NY USA; 3 Antitumor Assessment Core Facility, Memorial Sloan Kettering Cancer Center, New York, NY USA

**Keywords:** EGFR, HER2, Bispecific antibody, Pancreatic ductal adenocarcinoma

## Abstract

**Background:**

EGFR and/or HER2 expression in pancreatic cancers is correlated with poor prognoses. We generated homodimeric (EGFRxEGFR or HER2xHER2) and heterodimeric (EGFRxHER2) T cell-engaging bispecific antibodies (T-BsAbs) to direct polyclonal T cells to these antigens on pancreatic tumors.

**Methods:**

EGFR and HER2 T-BsAbs were constructed using the 2 + 2 IgG-[L]-scFv T-BsAbs format bearing two anti-CD3 scFvs attached to the light chains of an IgG to engage T cells while retaining bivalent binding to tumor antigens with both Fab arms. A Fab arm exchange strategy was used to generate EGFRxHER2 heterodimeric T-BsAb carrying one Fab specific for EGFR and one for HER2. EGFR and HER2 T-BsAbs were also heterodimerized with a CD33 control T-BsAb to generate ‘tumor-monovalent’ EGFRxCD33 and HER2xCD33 T-BsAbs. T-BsAb avidity for tumor cells was studied by flow cytometry, cytotoxicity by T-cell mediated ^51^Chromium release, and in vivo efficacy against cell line-derived xenografts (CDX) or patient-derived xenografts (PDX). Tumor infiltration by T cells transduced with luciferase reporter was quantified by bioluminescence.

**Results:**

The EGFRxEGFR, HER2xHER2, and EGFRxHER2 T-BsAbs demonstrated high avidity and T cell-mediated cytotoxicity against human pancreatic ductal adenocarcinoma (PDAC) cell lines in vitro with EC50s in the picomolar range (0.17pM to 18pM). They were highly efficient in driving human polyclonal T cells into subcutaneous PDAC xenografts and mediated potent T cell-mediated anti-tumor effects. Both EGFRxCD33 and HER2xCD33 tumor-monovalent T-BsAbs displayed substantially reduced avidity by SPR when compared to homodimeric EGFRxEGFR or HER2xHER2 T-BsAbs (∼150-fold and ∼6000-fold respectively), tumor binding by FACS (8.0-fold and 63.6-fold), and T-cell mediated cytotoxicity (7.7-fold and 47.2-fold), while showing no efficacy against CDX or PDX. However, if either EGFR or HER2 was removed from SW1990 by CRISPR-mediated knockout, the in vivo efficacy of heterodimeric EGFRxHER2 T-BsAb was lost.

**Conclusion:**

EGFR and HER2 were useful targets for driving T cell infiltration and tumor ablation. Two arm Fab binding to either one or both targets was critical for robust anti-tumor effect in vivo. By engaging both targets, EGFRxHER2 heterodimeric T-BsAb exhibited potent anti-tumor effects if CDX or PDX were EGFR^+^HER2^+^ double-positive with the potential to spare single-positive normal tissue.

**Supplementary Information:**

The online version contains supplementary material available at 10.1186/s13045-024-01538-5.

## Background

Pancreatic cancer remains one of the most lethal forms of cancer despite considerable therapeutic efforts [1]. As of 2020, pancreatic cancer represents the 3rd leading cause of cancer-related deaths despite representing only 3% of all new cancer diagnoses [2]. With a high stroma to tumor ratio, poor immune infiltration, and an aggressive propensity for metastasis, treatment strategies for pancreatic cancer have seen modest advancement since the establishment of gemcitabine as the standard of care more than 20 years ago [3, 4]. The NIH’s predicted 5-year survival rate for patients with pancreatic cancer is a discouraging 11.5%, a statistic that has remained largely unchanged for decades, loudly begging for more effective strategies [2].

EGFR and HER2 are highly expressed in many pancreatic ductal adenocarcinomas (PDAC) and correlate with poor prognoses [5, 6]. Tumors with both EGFR and HER2 over-expression can be accompanied by c-myc overexpression [7, 8], and AEG-1 expression [9, 10], associated with a more invasive phenotype. EGFR- and HER2-directed monoclonal antibodies have been well-tolerated but failed to exhibit meaningful clinical efficacy in the treatment of PDAC [11, 12]. A recent phase I clinical trial exploring the safety and efficacy of EGFR-directed chimeric antigen receptor T (CAR T) cells in metastatic pancreatic carcinoma reported partial response (PR) or stable disease (SD) in 12 out of 14 evaluable patients (4 PR and 8 SD), although the median progression-free survival was only 3 months [13]. Several patients experienced grade ≥ 3 adverse effects, including pleural effusion and pulmonary interstitial exudation toxicities [13], a possible side effect of EGFR-directed on-target/off-tumor responses [14]. Similarly, 6 out of 11 pancreatic cancer patients in a phase I trial responded to HER2-specific CAR T cell therapy (1 PR, 5 SD), though progression-free survival was similarly brief (4.8 months) [15]. As with the previous study, patients experienced adverse effects, though most were mild or moderate [15]. Both studies underscore a potential for EGFR- and HER2-directed T cell-based therapies in pancreatic cancer. Though antibody therapies directed at single receptors have been largely ineffective, the consistent expression of EGFR and HER2 in PDAC could be exploited to improve efficacy while reducing on-target/off-tumor dose limiting complications.

We have previously described several T cell-engaging bispecific antibodies (T-BsAbs) [16–19], including a HER2-specific T-BsAb that demonstrated exceptionally strong anti-tumor activity in vitro and in vivo [20–22]. We have also elucidated the significance of interdomain spacing and spatial configuration of the IgG-[L]-scFv format in driving anti-tumor responses in vivo [23]. Built on the IgG-[L]-scFv format, we generated homodimeric EGFR and HER2 T-BsAbs (referred to otherwise as EGFRxEGFR and HER2xHER2 T-BsAbs respectively) which utilize scFv domains fused to the C-termini of each light chain to engage CD3 on T cells while retaining bivalent binding of the tumor targets with both Fab arms.

EGFR is expressed in some healthy tissues including skin [24], where much of the clinical toxicity of antibody treatments are directed [25]. HER2 expression is generally much lower in normal tissues [26], and cardiotoxicity is the most frequent dose-limiting side effects associated with the HER2 monoclonal antibodies [27, 28]. Toxicities associated with both EGFR and HER2-directed antibody treatments are typically well-tolerated and resolve upon cessation of treatment. With minimal overlap in EGFR and HER2 antibody-mediated toxicities, and high frequencies of EGFR and HER co-expression in PDAC and other cancers, the development of therapies that target both proteins is appealing. Using a Fab arm exchange strategy [29], we additionally generated EGFRxHER2 heterodimeric T-BsAbs possessing two IgG half molecules, each specific for either EGFR or HER2. This novel T-BsAb platform takes advantage of differential avidity between tumors (that express elevated levels of both targets) and normal tissues (that either express one or weakly express both targets) to improve the specificity of EGFR- and HER-directed T-BsAbs to EGFR^+^HER2^+^ double-positive tumors.

In the present study, we characterize the biochemical and cytotoxic properties of EGFRxEGFR, HER2xHER2 and EGFRxHER2 T-BsAbs built on the IgG-[L]-scFv format. We describe their ability to drive T cells into CDX and PDX to effect potent anti-tumor effects. We demonstrate the superiority of tumor-specific bivalency in effectuating anti-tumor responses in vivo, contrasting strongly with ‘tumor-monovalent’ T-BsAbs, which exerted minimal to no in vivo anti-tumor effects. While the EGFRxHER2 heterodimer T-BsAb could ablate EGFR^+^HER2^+^ double-positive pancreatic tumors, deletion of either EGFR or HER2 completely abrogated antitumor efficacy. Heterodimerization of T-BsAbs has the potential to significantly reduce on-target/off-tumor side effects, i.e. to spare healthy single-positive tissues, by restricting their effects to double-positive tumor tissues.

## Materials and methods

### Bispecific antibody (T-BsAb) design and heterodimerization

The anti-EGFR T-BsAb and anti-HER2 T-BsAb were designed with VH/VL domains from corresponding humanized antibodies using an IgG-L-scFv format [16, 30] where huOKT3 scFv was fused to the C-terminus of the light chain of a human IgG1. To prevent glycosylation, Fc-receptor binding or complement activation, N297A and K322A mutations in the Fc region were introduced [31, 32].

T-BsAbs were expressed by and purified from HEK293T cells (RRID: CVCL_KS61) and dialyzed in 25mM sodium citrate buffer with 150mM NaCl (pH 8.2).

T-BsAbs were heterodimerized using a Fab Arm Exchange strategy [29]. 2-Mercaptoethanol was used to cleave inter-H-chain disulfide bonds. The cleaved BsAbs were subsequently dialyzed in citrate buffer, and amino acid substitutions F405L and K409R drove the cleaved products to heterodimerize during reformation.

### FACS analysis

Assessment of T-BsAb surface binding was performed by incubating target cells with the indicated concentrations of T-BsAbs for 45 min at 4°C. Cells were then washed and reacted with an R-phycoerythrin-conjugated goat anti-human IgG secondary antibody (SouthernBiotech Cat#2040-09, RRID: AB_2795648) and analyzed by flow cytometry (Attune NxT Flow Cytometer).

Characterization of intratumor lymphoid and myeloid utilized the following antibodies purchased from BioLegend: anti-human CD45-PE-Cy7 (Cat# 304,016), anti-human CD4-BV421 (Cat# 300,532), anti-human CD4-AF488 (Cat# 300,519), anti-human CD8-BV510 (Cat# 344,732), anti-human CD69-PE (Cat# 310,906), anti-human IFN-γ-APC (Cat# 502,512), anti-human TNF-α-FITC (Cat# 376,208), anti-mouse/human CD11b-PE-Cy7 (Cat# 101,215), anti-mouse PDL1-PerCP-Cy5.5 (Cat# 124,333).

### Cell lines and patient-derived xenografts

Human PDAC cell lines SW1990 (RRID: CVCL_1723), BxPC-3 (RRID: CVCL_0186), Capan-2 (RRID: CVCL_0026), CFPAC-1 (RRID: CVCL_1119), AsPC1 (RRID: CVCL_0152), PANC-1 (RRID: CVCL_0480), Panc 10.05 (RRID: CVCL_ 1639) were obtained from ATCC.

SW1990 EGFR-KO and SW1990 HER2-KO were generated from the parental SW1990 cell line using a CRISPR-Cas9 system with the assistance of the Gene Editing & Screening Core Facility at MSKCC.

One patient-derived tumor xenografts (PDXs) used in in vivo studies, 1aS1, was established from surgical specimens by the Antitumor Assessment Core Facility in MSKCC with MSKCC IRB approval.

Rh-41 cells (RRID: CVCL_2176) were provided by the Dr. R.J. O’Reilly at MSK and originally purchased from the Leibniz Institute DSMZ. TC32 cells (RRID: CVCL_7151) were obtained from ATCC. OSCA-40 (RRID: CVCL_L389) were purchased from Kerafast.

PDAC cells were cultured in RPMI1640 (Corning) with 10% fetal bovine serum (FBS, Life Technologies) at 37 °C in a 5% CO2 humidified incubator.

### Cytotoxicity assay (chromium^51^ release assay)

In vitro antibody-dependent T cell-mediated cytotoxicity (ADTC) assays were performed as described previously [19]. Target cells were labeled with ^51^Cr radionuclide (Perkin Elmer, Boston MA) at 100 µCi/10^6^ cells at 37 °C for 1 h with frequent resuspension. Specific lysis was calculated as a percentage using the formula 100 x ((experimental cpm) – (background cpm) / (maximum cpm – background cpm) where cpm represents counts per minute of ^51^Cr released. Maximum cpm was determined by lysis with 10% SDS (Siga, St Louis, Mo), and background cpm was measured in the absence of effector cells and ^51^Cr. Anti-CD33 T-BsAb was routinely used as a control T-BsAb.

### Surface plasmon resonance (SPR) analysis

Either recombinant human EGFR (R&D system: Cat# 9565-ER), HER2 (Sino Biological: Cat# 10,004-H08H), or both were immobilized on CM5 chips. Mixed EGFR and HER2 were immobilized using a ratio of 5:1 EGFR to HER2 to loosely replicate the ratio of EGFR and HER2 expression on PDAC cell lines as determined from MFIs from FACS analysis. T-BsAbs were flowed over the chip using a Biacore T200 system. Binding kinetics of the T-BsAbs were measured at 37°C. Data from HER2-immobilized chips were fitted with 1:1 model while data from EGFR-immobilized and mixed chips were fitted with two-state reaction model.

### T cell expansion ex vivo

Peripheral blood mononuclear cells (PBMCs) were separated from buffy coats (provided by New York Blood Center) by Ficoll. T cells were purified from human PBMC using a Pan T cell isolation kit (Miltenyi Biotec, Cat#130,096,535). T cells were expanded with CD3/CD28 Dynabeads (Gibco, Cat#11 132D) for 7–14 days in the presence of 30 IU/mL of IL- 2 (PROLEUKIN, Prometheus Laboratories Inc.).

### In vivo studies

All mouse experiments were performed in compliance with the Institutional Animal Care and Use Committee guidelines. Immunodeficient BALB/c IL-2rg^−/−^, Rag2^−/−^ (BRG, 11,503) mice were purchased from Taconic [33] and provided with Sulfatrim food. For tumor challenge experiments, PDAC cells were suspended in Matrigel Basement Membrane Matrix (Corning, Cat#354,234) and implanted subcutaneously in the flank of 6- to 10-week-old mice (2-3 × 10^6^ cells/mouse). Tumors were measured using a TM900 scanner (Piera, Brussels, BE). After ∼18 days (tumors approximately 100-200mm^3^), mice were administered T-BsAb (twice weekly) and human activated T cells (once weekly) by retro-orbital injection. T cells were expanded in culture for 7–9 days before injection on the same day as T-BsAbs. BRG mice lack T and B cells and do not reject infusions of human T cells, which can be engaged by anti-CD3 scFv domains (OKT3) of the T-BsAbs. 1000IU of interleukin-2 (IL2) was subcutaneously injected 2 times per week to support T cell persistence. To define well-being of mice, changes in body weight, general activity, physical appearance, and graft-versus-host disease (GVHD) scoring were regularly monitored. Mice with tumors exceeding 2000mm^3^ were euthanized. Control groups included mice that received no T cells or T-BsAbs, or mice that received T cells-only. All in vivo experiments included a second control group that received T cells plus CD33xCD33 T-BsAbs, chosen because human CD33 is expressed in neither the infused human T cells nor PDAC xenografts, and were compared with treatment groups receiving T cells, and either 2 µg or 10ug of T-BsAbs. 5 mice per group were injected with tumors and involved in analyses unless stated otherwise. This number is based on previous experiments conducted using T-BsAbs in our lab that suggest this sample size is sufficiently large to determine statistically significant differences in treatment efficacy. Not all patient-derived xenografts successfully implanted. Mice without established tumors were excluded from the experiment, resulting in *n* = 4 for each group. All tumor-bearing mice were randomized into control and treatment groups and were included in the analyses. All cages within an experiment were housed together on the same rack in the same room of MSKCC’s RARC. The order of treatments, measuring and imaging varied from week to week to reduce potential confounding. Blinding was not possible because one person was responsible for the execution and analysis of these experiments.

### Immunohistochemistry staining and PDAC PDX samples

Immunohistochemical analysis of OCT-embedded tumors was performed using a previously-described methodology [34]. Tissues were stained using 2 µg/ml of T-BsAbs.

22 paraffin-embedded patient-derived xenograft pancreatic cancer tissues were provided by the MSK Antitumor Assessment Core Facility. Tissues were sectioned and immunohistochemical staining was performed by the MSK Molecular Cytology Core Facility as previously described [16].

All images were captured from prepared slides using a Nikon ECLIPSE Ni-U microscope and NIS-Elements imaging software. EGFR and HER2 staining were analyzed and interpreted by a pathologist using guidelines described by Atkins et al. [35] and Wolff et al. [36], respectively.

### Pharmacokinetic studies

BRG mice (*n* = 5/group) were serially bled from 0.5 to 168 h after intravenous T-BsAb administration (100 µg). Plasma concentrations of BsAb were determined by ELISA using the method previously described [23].

### Depleting antibodies

100 µg of anti-mouse GR-1 (Bio X Cell Cat# BE0320, RRID: AB_2819047) or 100 µg of anti-mouse CSF-1R (CD115) antibody (Bio X Cell Cat# BE0213, RRID: AB_2687699) were administered by i.p. injection twice a week to deplete granulocytes and monocytes as previously reported [37].

### T cell transduction with tdTomato and click beetle red luciferase

Similar to the T cell expansion protocol described earlier, T cells were isolated from peripheral blood (PB) and stimulated with Dynabeads™ Human T-Activating CD3/CD28 beads (GibcoTM, Cat#11132D) for 24 h. T cells were then transduced with retroviral plasmids containing tdTomato and click beetle red luciferase in RetroNectin-coated 6-well plates with IL-2 (100IU/ml) and protamine sulfate (4 µg/mL). Luciferase-transduced T cells (Luc(+) T cells) were expanded in culture for 7–9 days prior to infusion. In all in vivo studies involving Luc(+) T cells, only the first infusion of T cells were Luc(+). All subsequent infusions used non-transduced T cells.

### T cell trafficking and bioluminescence imaging

Mice were administered 3 mg of D-luciferin (Gold Biotechnology, Cat# LUCK-100) by retro-orbital injection prior to anesthetization with isoflurane and imaging. Bioluminescent images were acquired using an IVIS Spectrum CT In vivo Imaging System (Caliper Life Sciences) hosted by the MSK Animal Imaging Core Facility. Regions of interest (ROI) were drawn based on the location and contour of tumor using Living image 2.60 (Xenogen) to quantify bioluminescence emission (BLI, photon flux/sec).

### Statistical analyses

Tumor growth rates and bioluminescence of T cells were analyzed using area under the curve (AUC). The AUC and overall survival were calculated using GraphPad Prism. Differences between treatment groups were tested for statistical significance by two-tailed Student’s t-test (comparing two sets of data) and one-way ANOVA with Tukey’s post hoc test (for three or more data sets). All statistical analyses were performed using GraphPad Prism (GraphPad Software, La Jolla, CA). P value < 0.05 was considered statistically significant. Asterisks indicate that the experimental P value is statistically significantly different from the associated controls; * *P* < 0.05; ** *P* < 0.01; *** *P* < 0.001, **** *P* < 0.0001.

## Results

### T-BsAb design and target evaluation in human PDAC cell lines and patient-derived xenografts

**Fig. 1:**
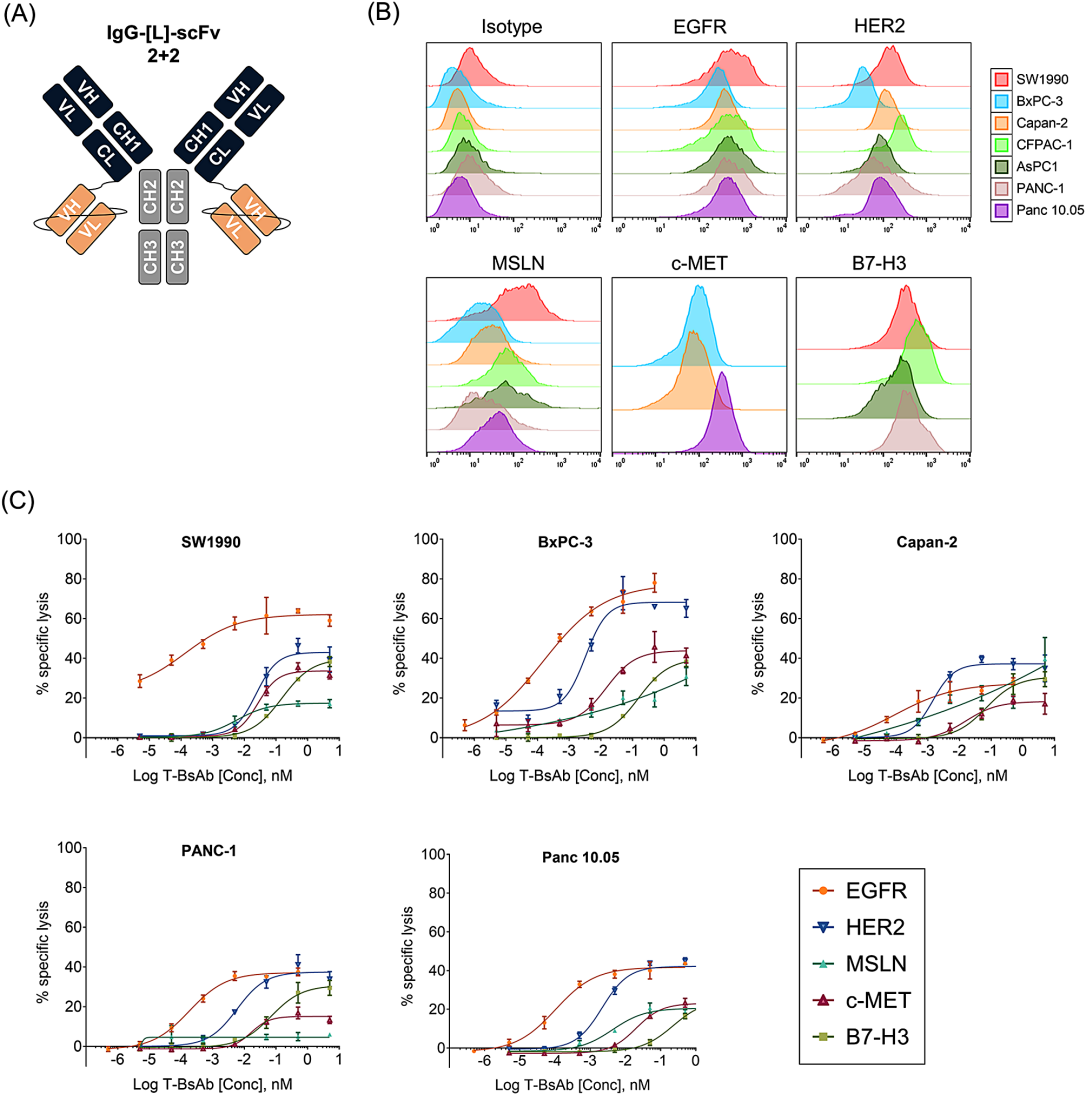
Structure and in vitro activity of PDAC-directed T cell-engaging bispecific antibodies (**A**) IgG-[L]-scFv structure of BsAbs. CH, constant heavy chain; CL, constant light chain; scFv, single chain variable fragment; VH, variable heavy chain; VL, variabl light chain. (**B**) Flow cytometric analysis displaying fluorescent intensities of IgG-[L]-scFv BsAbs bound to human PDAC cells. IgG-[L]-scFv BsAbs evaluated were specific for human EGFR, HER2, MSLN, c-MET, and B7-H3. (**C**) Antibody-dependent T cell-mediated cytotoxicity (ADTC) as a function of increasing doses of BsAbs against PDAC cell lines. Ratio of effector T cells to target PDAC cells (E: T ratio) was set to 10:1

EGFR-, HER2-, MSLN-, c-MET-, and B7-H3-directed T cell-engaging bispecific antibodies (T-BsAbs) were generated using the IgG-[L]-scFv platform for evaluation against PDAC cell lines (Fig. 1A). The platform utilizes an anti-CD3 humanized OKT3 (huOKT3) scFv fused to the C-terminus of the light chain to engage CD3 on T cells. The T-BsAbs demonstrated appreciable binding to all PDAC cell lines tested (SW1990, BxPC-3, Capan-2, PANC-1, PANC 10.05) by flow cytometry, with EGFR T-BsAb consistently among the highest mean fluorescent intensities against all cell lines (Fig. 1B; Table 1).

**Table 1:** Tumor-associated antigen expression (normalized MFI, mean fluorescence intensity) in PDAC cell lines

Cell line	EGFR	HER2	MSLN	c-MET	B7-H3
SW1990	39.1	10.3	9.2		25.2
BxPC3	48.5	6.3	1.8		
Capan 2	60.6	14.0	5.9	47.0	
CFPAC-1	53.9	30.8	9.3		76.0
AsPC1	44.7	8.1	6.2		17.9
Panc1	65.9	21.4	5.2	12.3	32.4
Panc10.05	37.7	5.7	2.9	14.4	

EGFR, epidermal growth factor receptor; HER2, human epidermal growth factor receptor 2; MSLN, mesothelin; c-MET, tyrosine-protein kinase Met; B7-H3, B7 homolog protein 3, CD276

We next evaluated the capacity of the T-BsAbs to drive antibody-dependent T cell-mediated cytotoxicity against the panel of PDAC cell lines, with potency measured by EC50. EGFR T-BsAb consistently demonstrated lowest EC50s in the femtomolar range (9-18fM) across all cell lines (Fig. 1C; Table 2). All other T-BsAbs consistently yielded EC50s in the picomolar range with HER2 T-BsAb frequently exhibiting the second lowest EC50s (1.44-23.55pM) after EGFR.

**Table 2:** In vitro sensitivities (EC50, pM) to target antigen-specific bispecific antibodies in PDAC cell lines

Cell line	EGFR	HER2	MSLN	c-MET	B7-H3
SW1990	0.1	23.6	5.8	26.7	155.4
BxPC-3	0.2	3.3	ND	14.9	
Capan-2	0.1	1.4	ND	15.6	70.0
PANC-1	0.2	5.9	ND	16.3	
Panc 10.05	0.1	2.2	4.3	20.2	227.1

EGFR, epidermal growth factor receptor; HER2, human epidermal growth factor receptor 2; MSLN, mesothelin; c-MET, tyrosine-protein kinase Met; B7-H3, B7 homolog protein 3, CD276

In some instances, cell lines with lower expression of EGFR or HER2 yielded lower or comparable EC50s of T-BsAb-mediated cytotoxicity. For example, PANC-1 cells exhibited higher normalized EGFR and HER2 MFIs than Panc 10.05 (65.9 and 37.7 respectively) but displayed a higher EC50 (0.18pM and 0.10pM). Discrepancies like these may be intrinsic to cell lines, with some cell lines displaying greater innate susceptibility or resistance to T cell mediated cytotoxicity. Comparing results within each cell line, however, all cell lines with higher expression of EGFR than HER2 correspondingly exhibited lower EC50s to EGFR T-BsAb-mediated lysis compared to HER2 T-BsAbs.

We evaluated a panel of 22 human PDAC PDXs for EGFR and HER expression by immunohistochemical analysis (Supp. Table 1). EGFR expression was detected in 15 samples (72.7%) with 5 samples (22.7%) exhibiting moderate expression or higher (score ≥ 2). HER2 was detected in 6 (27.3%) and moderate to high expression was observed in 2 samples (9.1%). 5 out of 6 samples with detectable HER2 co-expressed EGFR (22.7%).

### EGFR and HER2 heterodimeric T-BsAbs target PDAC

**Fig. 2:**
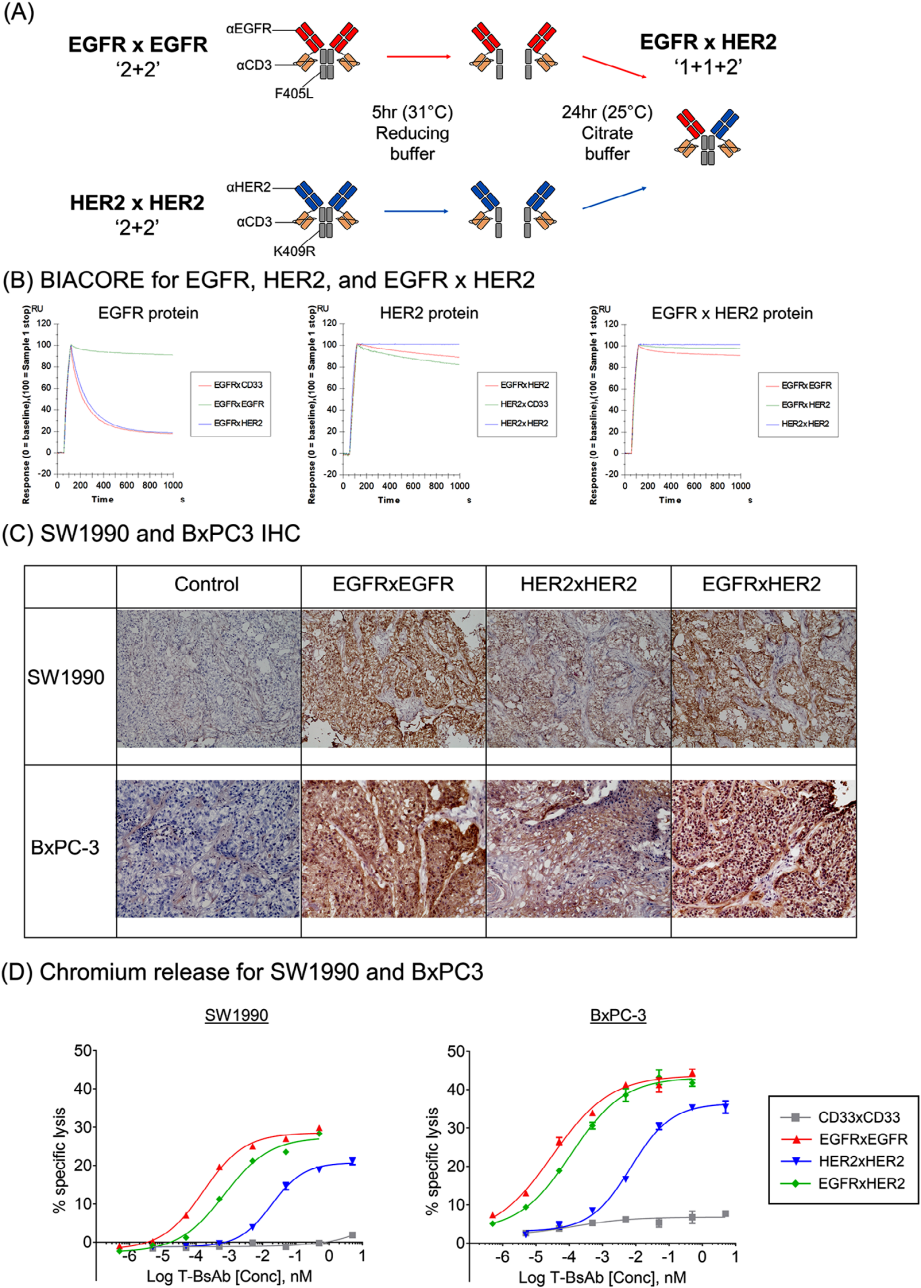
EGFR and HER2 T-BsAb heterodimerization and in vitro kinetics (**A**) Schematic of BsAb heterodimerization by controlled Fab Arm Exchange. Parental BsAbs bore 2 Fabs specific to EGFR or HER2 and 2 scFvs specific for CD3 (‘2 + 2’). Reduction of inter-H-chain disulfide bonds and subsequent heterodimerization (driven by F405L and K409R amino acid substitutions) yielded EGFRxHER2 BsAbs bearing 1 EGFR Fab, 1 HER2 Fab, and 2 CD3 scFv (‘1 + 1 + 2’). (**B**) SPR analysis of T-BsAbs binding to EGFR (left panel), HER2 (middle panel), and EGFR x HER2 (right panel) proteins. Representative normalized sensorgrams at 20nM were shown. (**C**) IHC staining of two cell-derived PDAC xenograft tumors SW1990 (top) and BxPC-3 (bottom). OCT-embedded tumor sections were stained with EGFRxEGFR, HER2xHER2, or EGFRxHER2 BsAbs. Control slides were either unstained (SW1990) or incubated with a control antibody (BxPC-3). (**D**) Antibody-dependent T cell-mediated cytotoxicity assays (ADTC) against SW1990 (left) and BxPC-3 (right). Cytotoxicity measured by Chromium-51 release. Ratio of effector T cells to target PDAC cells (E: T ratio) was set to 10:1. EC50s (SW1990; BxPC-3): EGFRxEGFR: 165.8fM; 31.5fM, HER2xHER2: 18.5pM; 7.14pM, EGFRxHER2: 699.6fM; 128.8fM. CD33xCD33 BsAb was included as a negative control

The homodimeric EGFR and HER2 T-BsAbs were built using the cetuximab and trastuzumab amino acid sequences, respectively. Based on the high frequency of EGFR and HER2 co-expression on PDAC cells and tumors, EGFRxEGFR and HER2xHER2 T-BsAbs were chosen to generate a heterodimeric T-BsAb using a controlled Fab Arm Exchange (cFAE) strategy (Fig. 2A). The resulting EGFRxHER2 heterodimeric BsAb possesses one Fab specific for EGFR and one for HER2 while retaining two anti-CD3 scFvs. A similar process was used to additionally heterodimerize EGFR and HER2 BsAbs with an irrelevant CD33 BsAb. The EGFRxHER2 heterodimeric BsAb demonstrated a significant reduction in avidity to EGFR or HER2 protein-coated chips compared to the homodimeric counterparts, determined by surface plasmon resonance (Fig. 2B; Table 3). When applied to a chip coated with both EGFR and HER2, the avidity of the EGFRxHER2 T-BsAb improved to levels comparable to the homodimeric T-BsAbs.

**Table 3:** Avidities of EGFR and HER2 T-BsAbs

Specificity	Chip-bound protein	Binding valency	K_D_ (M)
EGFR x EGFR	EGFR	2	1.33E-11
EGFR x HER2	EGFR	1	1.76E-09
EGFR x CD33	EGFR	1	1.96E-09
HER2 x HER2	HER2	2	1.12E-13
EGFR x HER2	HER2	1	2.75E-10
HER2 x CD33	HER2	1	6.65E-10
EGFR x EGFR	EGFR + HER2	2	1.42E-11
HER2 x HER2	EGFR + HER2	2	6.18E-13
EGFR x HER2	EGFR + HER2	2	6.04E-12

Immunohistochemical analysis of SW1990 and BxPC-3 tumors revealed strong staining of tumor tissues by all three EGFRxEGFR, HER2xHER2, and EGFRxHER2 T-BsAbs. EGFRxEGFR and EGFRxHER2 T-BsAbs demonstrated the strongest staining and were comparable in intensity (Fig. 2C). In vitro cytotoxicity assays similarly demonstrated potent killing by EGFRxHER2 T-BsAbs, although EC50s were 3.2-4.6-fold higher than EGFRxEGFR homodimers, yet 26.4-62.0-fold lower than HER2xHER2 homodimers (Fig. 2D).

### EGFR and HER2 T-BsAbs effectively treat PDAC xenografts

**Fig. 3:**
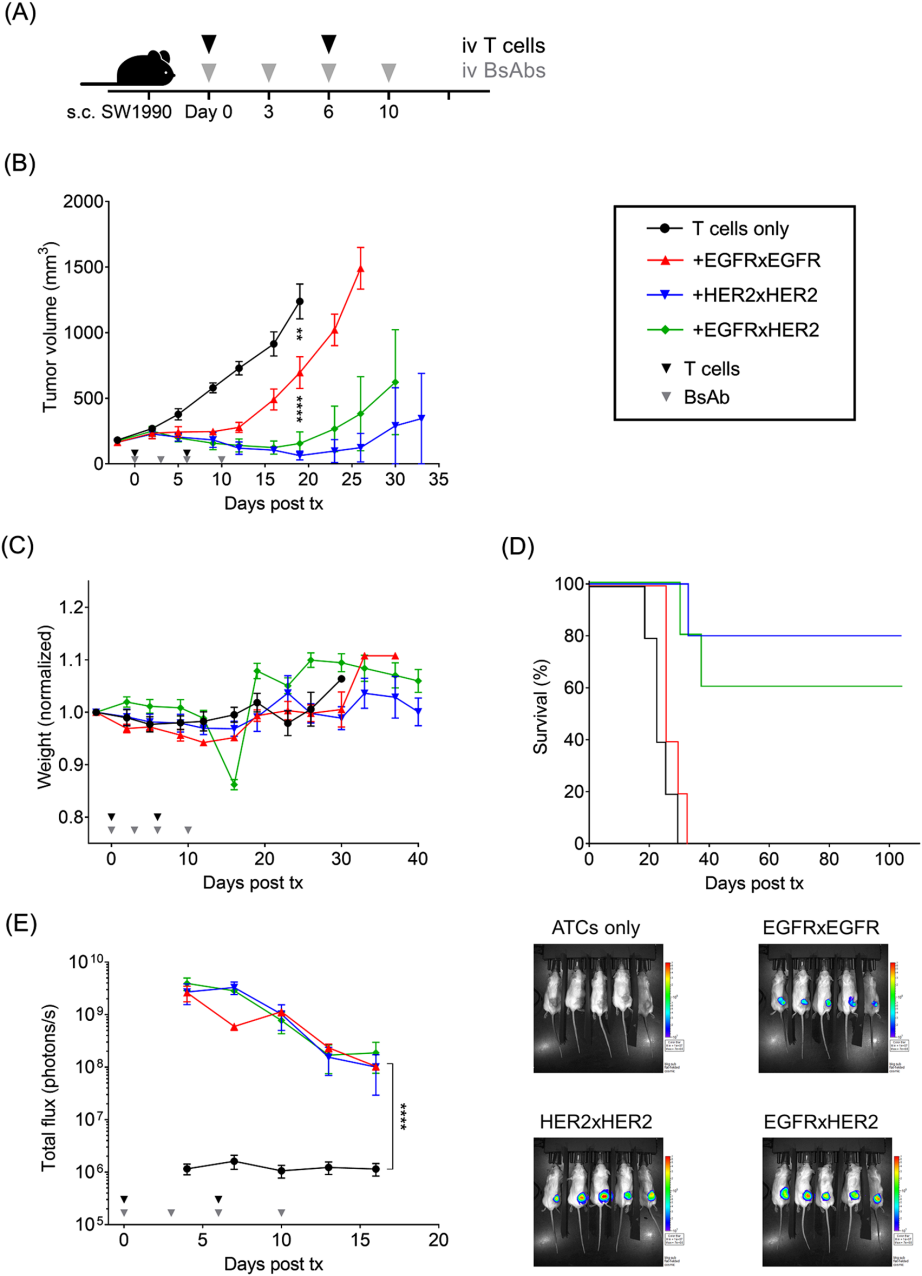
Heterodimeric EGFR and HER2 T-BsAbs impede PDAC growth (**A**) In vivo antitumor effect of BsAbs in the presence of human T cells against SW1990 cell line xenografts; 3 × 10^6^ cells of SW1990 were subcutaneously implanted into mice. T-BsAbs were administered twice per week and 2 × 10^7^ of luciferase-transduced T cells (Luc(+) T cells) were administered once per week for 2 weeks to treat the tumors. (**B**) In vivo anti-tumor effects of 10 µg EGFR and HER2 T-BsAbs. (**C**) Relative body weight of mice during treatment and (**D**) overall survival were plotted. (**E**) Bioluminescence imaging (BLI) of Luc(+) T cells. Quantitation of bioluminescence intensity in the lesions of tumors. Representative images (right) were taken on day 7

In vivo efficacies of EGFRxEGFR, HER2xHER2, and EGFRxHER2 T-BsAbs were evaluated against SW1990 PDAC xenografts. Mice were treated twice a week with 10 µg doses of T-BsAbs and given infusions of human T cells once per week for two weeks (Fig. 3A). All three T-BsAbs demonstrated efficacy (Fig. 3B). All mice tolerated treatment well with no signs of weight loss (Fig. 3C) or GVHD. On day 16, all mice in the EGFRxHER2 T-BsAb-treated group exhibited sudden weight loss. All mice recovered their weight without intervention 3 days later. Considering the mouse were housed in the same cage and showed no other signs of toxicity, this spontaneous and transient weight loss is ascribed to a water bottle malfunction. Interestingly, despite superior results in vitro, homodimeric EGFR T-BsAb treatment resulted in the poorest control of tumor growth and offered no significant benefit to survival (Fig. 3D). Conversely, both HER2 and EGFRxHER2 T-BsAbs showed impressive tumor control and improved survival, with several mice achieving remission. In the BxPC-3 PDAC xenograft model, T-BsAb treatments demonstrated similar effects on tumor growth, although no mice achieved complete remission (*n* = 4) (Supp. Figure 1). Despite differences in efficacy in SW1990 tumors, bioluminescent imaging of T cells revealed all T-BsAb treatments promoted T cell homing to tumors (Fig. 3E). Due to the low TIL frequencies of the ‘ATCs only group’—and correspondingly low flux values in tumors of the ‘ATCs only’ mice compared to T-BsAb-treated mice—the representative image appears ‘dark’ when uniform bioluminescence intensity scaling was used for all groups.

Anti-EGFR and anti-HER2 monospecific antibodies (specifically cetuximab and trastuzumab which our T-BsAbs’ variable domain sequences are based on) have been shown to target exclusively to xenograft tumors bearing the cognate antigens [38]. Unlike the pharmacokinetics (PK) of monospecific IgG which reacts with its single cognate antigen, the PK of bispecific antibodies is determined by the temporal and spatial interactions of two specificities [39], where each specificity has its own target sink and unique in vivo biodistribution. In our tumor models, xenografts are immunologically cold, even after infusion with human T lymphocytes. When injected i.v., T-BsAb of 150kD-200kD size will take hours to penetrate the xenograft. But within minutes inside the intravascular space, T-BsAb will be sequestered by the circulating hhuman T cells which exist in vast numbers. Even for small tissue-penetrating T-BsAbs such as BiTEs, its biodistribution can be heavily driven by its anti-CD3 arm [40]. Most published biodistribution studies using monovalent T-BsAb (BiTE or heterodimeric T-BsAb) showed lymphoid uptake to varying degrees. With the 2 + 2 bivalent format, the administered T-BsAb became T-cell bound with variable amounts of free T-BsAb left to traffic as free antibodies to the tumor. Since T-BsAbs are T-cell bound, T-BsAb biodistribution can not be meaningfully studied; it can only be indirectly evaluated by following/tracking T cells, which we assessed in a luciferase-transduced T cell (Luc(+) T cell) reporter assay (Fig. 3E). In Fig. 3, based on the bioluminescence overtime, EGFRxEGFR, HER2xHER2, and EGFRxHER2 T-BsAbs demonstrated comparable efficiency in driving T cells exclusively into the tumors. As early as day 4, all three T-BsAb treatments elicited ∼3000-fold greater photon flux from intratumoral Luc(+) T cells than mice receiving Luc(+) T cells only.

Although initial T-BsAb-driven intratumoral T cell homing was similar among the three T-BsAbs, their persistence within tumors could differ. When bioluminence was integrated over time (AUC) there was a significant reduction (*p* < 0.05, one-way ANOVA with Tukey’s post hoc test on log-transformed AUC values including only the three T-BsAb treatment groups) in AUC between HER2xHER2 and EGFRxEGFR as well as between EGFRxHER2 and EGFRxEGFR, but not between HER2xHER2 and EGFRxHER2. Significance was demonstrated for three T-BsAb doses, namely 0.4µg, 2µg, and 10µg but again none between EGFRxHER2 and HER2xHER2. We have included additional bioluminescence curves and their images over time following Luc(+) T cell and T-BsAb infusions for each of these doses (Supp. Figure 2). Beyond day 10, the flux values were too low to visualize on the same scale used for earlier time points. This diminished signal could be the result of T cell death, insufficient proliferation, and/or T cell departure, as a result of antibody affinities and TIL biology, mechanisms that require in depth analysis beyond the scope of this manuscript. As expected, this diminished TIL signal was associated with inferior antitumor effects against SW1990 tumors compared to HER2xHER2 and EGFRxHER2 T-BsAbs (Fig. 3B).

The pharmacokinetics (PK) of these three T-BsAbs in tumor-free mice were similar (Supp. Figure 3).

### T-BsAb treatments promote T cell infiltration, activation, and function

**Fig. 4:**
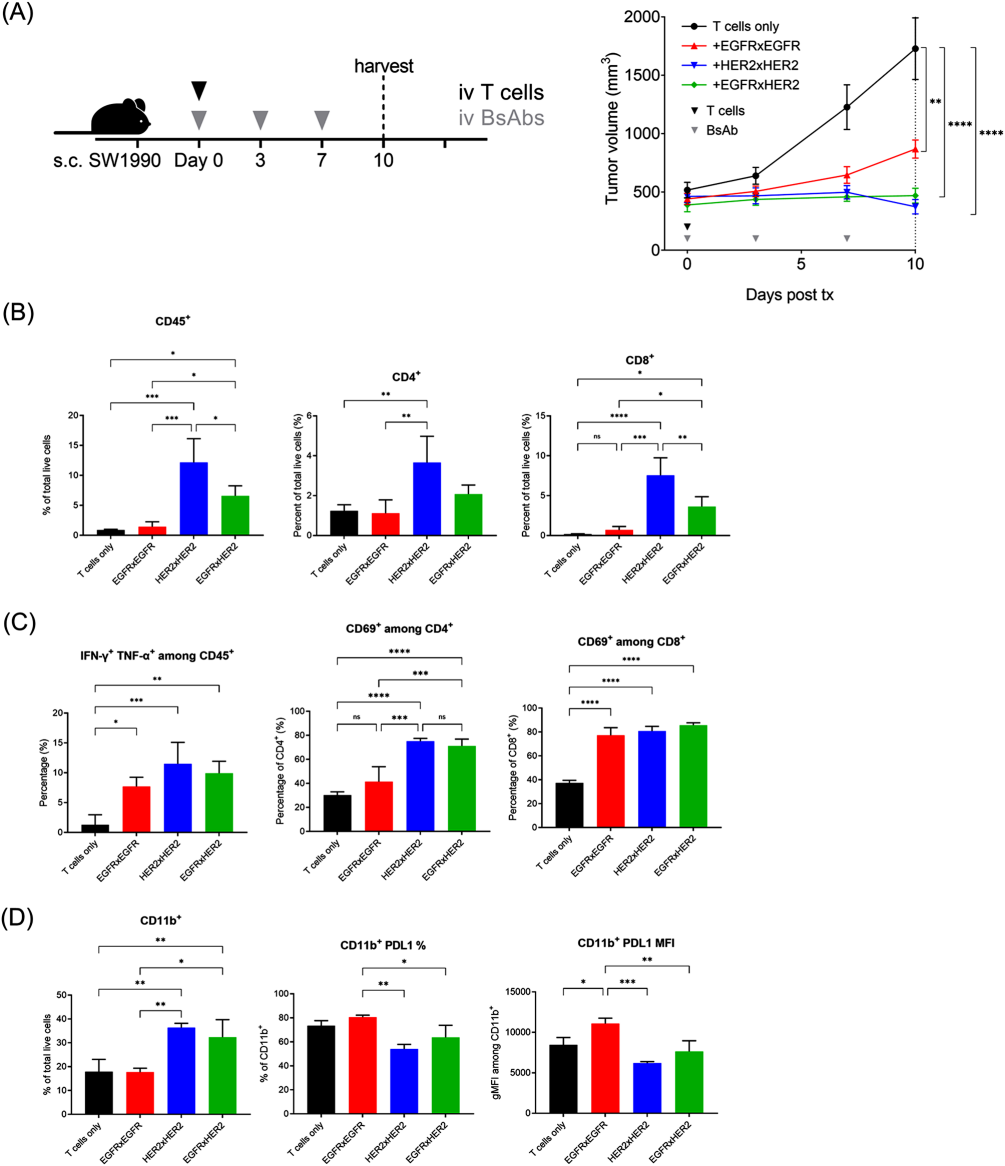
T-BsAbs drive T cell infiltration, activation, and function in PDAC (**A**) Treatment of SW1990 tumor-bearing mice with EGFR and HER2 T-BsAbs; 3 × 10^6^ cells of SW1990 were subcutaneously implanted into mice. Once tumor reached ∼500mm^3^, mice received a single infusion of 2 × 10^7^ T cells and were treated with 10µg T-BsAb, administered twice per week. Tumors were harvested for analysis on day 10 after initiation of treatment. Mice that exhibited 50% or more reduction in tumor volume by day 10 were excluded from this and subsequent analyses. (**B**) Flow cytometric analysis of the in vivo effect of T-BsAb treatments on T cell infiltration into harvested SW1990 PDAC tumors. Infiltrating T cells were identified by detection of human CD45^+^ and human CD4^+^ or CD8^+^. (**C**) Frequency of T cell activation indicated by detection of IFN-γ^+^TNF-α^+^ T cells and expression of CD69 among CD4^+^ or CD8^+^ T cells. (**D**) Frequency of CD11b^+^ intratumoral myeloid cells and prevalence of PDL1 expression

We further examined the impact of T-BsAb treatment on the functional status of TILs as well as the tumor microenvironment during early stages of treatment responses. SW1990 tumor-bearing mice were given one infusion of human T cells and treated with 10ug EGFR, HER2, and EGFRxHER2 T-BsAbs every 3–4 days until tumors were harvested 10 days after the start of treatment (Fig. 4A). As the focus of this study is to exam the effects of T-BsAb treatment at early stages antitumor efficacy, mice that demonstrated greater than 50% reduction in tumor size by day 10 following initiation of treatment were excluded from analyses. This criterion resulted in the exclusion of one HER2xHER2 T-BsAb-treated mouse that exhibited a 75% reduction in tumor volume. Similar to previous in vivo experiments, all T-BsAb treatments elicited significant delays in tumor growth, and although differences between treatments were beginning to emerge at the time tumors were harvested on day 10, the differences were not statistically significant.

Flow analysis of tumors revealed a higher proportion of human CD45^+^ cells in HER2 and EGFRxHER2 T-BsAb-treated tumors, representing greater presence of TILs. HER2xHER2 T-BsAbs elicited greater T cell infiltration than EGFRxHER2 T-BsAbs (Fig. 4B), while EGFRxEGFR T-BsAb treatment showed no significant increase in TILs compared to untreated tumors. T-BsAb treatment also increased the activation and cytokine production of TILs (Fig. 4C). All T-BsAb treatments increased the proportion of IFN-γ^+^TNF-α^+^ among human CD45^+^ T cells. All three T-BsAb treatments also elevated the expression of activation marker CD69 among both CD4^+^ and CD8^+^ TILs, although EGFR T-BsAb treatment did not significantly increase CD69 among CD4^+^ TILs.

Interestingly, the proportion of CD11b^+^ myeloid cells was significantly increased in HER2xHER2 and EGFRxHER2 T-BsAb-treated tumors. Across all groups, the vast majority of CD11b^+^ myeloid cells expressed PDL1. However, EGFRxEGFR T-BsAb treatment elicited a higher percentage and MFI of PDL1 expression compared to HER2 and EGFRxHER2 T-BsAbs, indicative of a more immunosuppressive myeloid phenotype. Although PDL1 expression was apparent in large proportions of CD11b^+^ myeloid cells across all groups, EGFRxEGFR T-BsAb-treated tumors exhibited a significantly higher percentage and stronger MFI of PDL1 expression among CD11b^+^ cells, consistent with a more immunosuppressive myeloid phenotype.

### Depletion of myeloid cells improves T-BsAb treatment

Pancreatic cancers are known for being one of the most ‘stroma rich’ tumors. The majority of PDAC tumors are comprised of stromal cells such as fibroblasts and myeloid cells [41]. Examination of SW1990 tumors revealed a significant and potentially immunosuppressive myeloid compartment, warranting further exploration of the contribution of myeloid cells on the poorer efficacy of EGFRxEGFR T-BsAb treatment. Myeloid-depleting antibodies were combined with EGFR T-BsAb treatment (Supp. Figure 4A). Anti-Gr-1 or anti-CSF-1R depletion antibodies were administered to deplete granulocytes and macrophages respectively. We have previously reported on the successful depletion effects of these antibodies [37]. Both depletion antibodies significantly delayed tumor growth in combination with EGFRxEGFR T-BsAbs compared to T-BsAb treatment alone and improved survival (Supp. Figure 4A and B). All treatments were well-tolerated (Supp. Figure 4D). Anti-Gr-1 and anti-GR-1 depletion antibodies improved T cell homing to tumors (Supp. Figure 4E).

### EGFR and HER2 T-BsAbs require bivalent binding for effective treatment

**Fig. 5:**
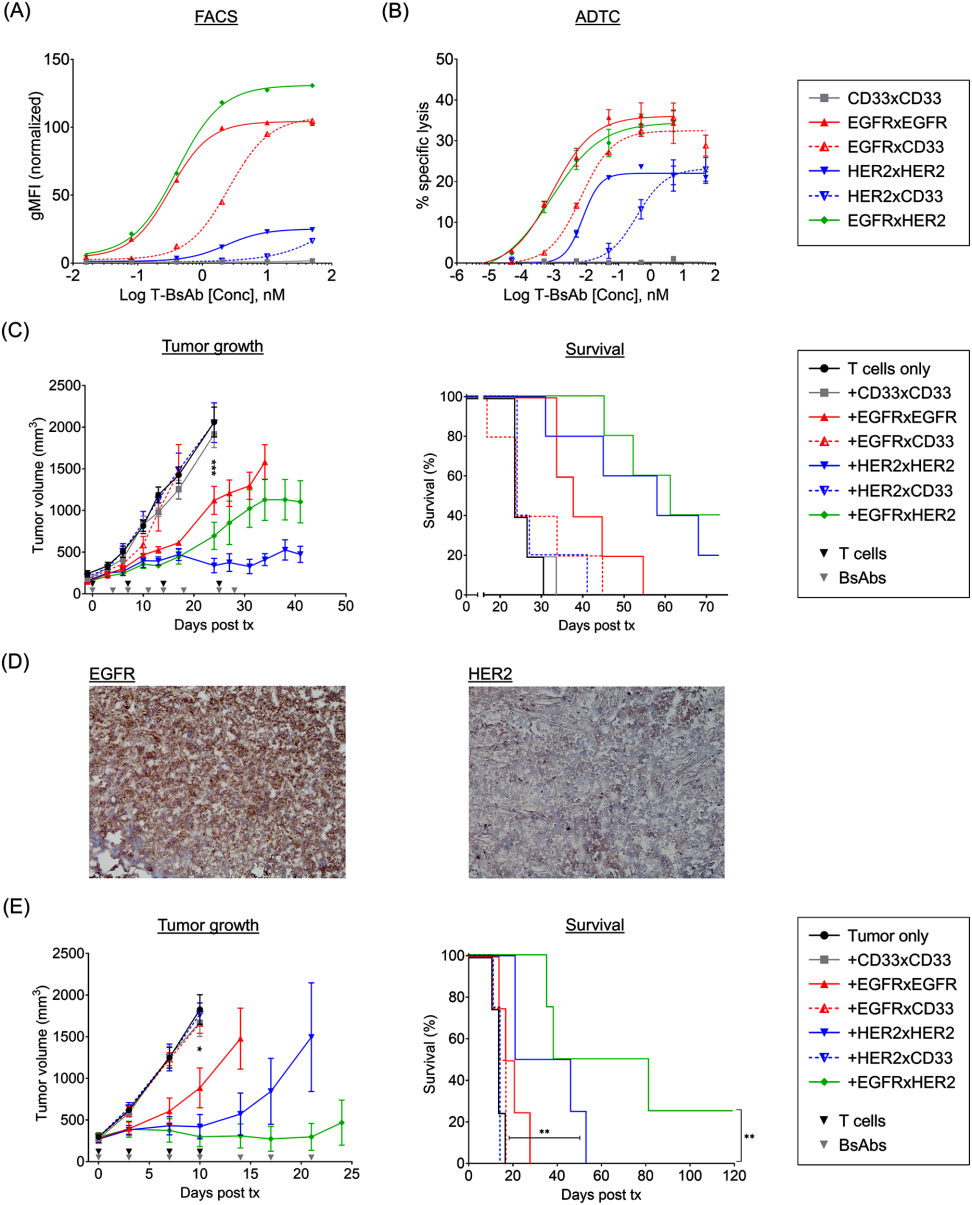
Reductions in tumor-binding valency critically impair T-BsAb efficacy in vitro and in vivo (**A**) Geometric mean fluorescent intensity of T-BsAbs against SW1990 cells measured by FACS. (**B**) Antibody-dependent T cell-mediated cytotoxicity (ADTC) assays targeting SW1990 cells. (**C**) Tumor growth of subcutaneous SW1990 xenograft (left) and survival (right). 2 × 10^7^ T cells were administered per infusion. 10µg T-BsAbs were administered i.v. twice a week. (**D**) IHC of 1aS1 tumors. (**E**) Tumor growth of subcutaneous 1aS1 patient-derived PDAC xenograft (left) and survival (right). 2 × 10^7^ T cells were administered per infusion. 2µg T-BsAbs were administered i.v. twice a week (*n* = 4 mice per group)

The EGFRxEGFR, HER2xHER2, and EGFRxHER2 T-BsAbs possess two tumor-specific Fabs that allow bivalent binding to EGFR^+^HER2^+^ double-positive PDAC tumors. We examined differences in ‘tumor monovalency’ and ‘tumor bivalency’ on binding strength and efficacy. Homodimeric EGFRxEGFR and HER2xHER2 T-BsAbs were heterodimerized with a CD33-specific control T-BsAb, generating EGFRxCD33 and HER2xCD33 T-BsAbs bearing one tumor-targeting Fab and one non-tumor-targeting CD33 control Fab. SPR analysis revealed significant reductions in avidity between EGFRxEGFR and EGFRxCD33 (∼150-fold) and between HER2xHER2 and HER2xCD33 (∼6000-fold) (Table 3). FACS analysis of mean fluorescent intensities revealed modest reductions in cell surface binding strength between the homodimeric bivalent T-BsAbs and their monovalent heterodimeric counterparts (8.0-fold degradation of EC50 from EGFRxEGFR to EGFRxCD33; 63.6-fold from HER2xHER2 to HER2xCD33) (Fig. 5A; Table 4). ADTC assays against SW1990 similarly revealed the loss of potency in monovalent T-BsAbs when compared to bivalent T-BsAbs (7.7-fold degradation of EC50 from EGFRxEGFR to EGFRxCD33; 47.2-fold from HER2xHER2 to HER2xCD33) (Fig. 5B; Table 5). Interestingly, in both FACS and ADTC analyses, the EGFRxHER2 heterodimer achieved EC50s similar to those of bivalent EGFRxEGFR T-BsAbs, which was the most effective T-BsAb in both in vitro assays.

**Table 4:** FACS binding (EC50, pM) of EGFR and HER2 T-BsAbs to SW1990 lines

Cell line	CD33xCD33	EGFRxEGFR	EGFRxCD33	HER2xHER2	HER2xCD33	EGFRxHER2
SW1990	—	319	2554	2302	146,300	402
SW1990 EGFR-KO	—	—	—	3363	46,935	31,475
SW1990 HER2-KO	—	280	4358	—	—	2224

We next investigated how the observed differences between tumor bivalent and tumor monovalent T-BsAbs would translate to efficacy in vivo. As in previous in vivo experiments, EGFRxEGFR, HER2xHER2, and EGFRxHER2 T-BsAb reduced SW1990 tumor growth, although only HER2xHER2 and EGFRxHER2 significantly prolonged survival (Fig. 5C). Strikingly, neither of the tumor monovalent T-BsAbs (EGFRxCD33 and HER2xCD33) demonstrated any benefit over ‘T cells only’ or CD33xCD33-treated control groups.

**Table 5:** ADTC sensitivities (EC50, pM) of SW1990 lines to EGFR and HER2 T-BsAbs

Cell line	CD33xCD33	EGFRxEGFR	EGFRxCD33	HER2xHER2	HER2xCD33	EGFRxHER2
SW1990	—	0.9	6.9	7.8	367.3	0.8
SW1990 EGFR-KO	—	—	—	38.3	557.5	229.5
SW1990 HER2-KO	—	0.2	6.4	—	—	8.2

A similar study was conducted in a patient-derived PDAC xenograft model ‘1aS1’. This aggressive model was derived from a metastasis after disease progressed to stage IV and after receiving multiple rounds of prior treatment. IHC revealed the 1aS1 xenograft to express high levels of EGFR and low levels of HER2 (Fig. 5D), consistent with the human PDAC cell models assessed earlier (Fig. 1B). Bivalently-engaging T-BsAbs again demonstrated appreciable control over 1aS1 tumor growth (*n* = 4) (Fig. 5E). Interestingly, EGFRxHER2 T-BsAb treatment appeared to exhibit better tumor control than either EGFRxEGFR or HER2xHER2 T-BsAbs. Similar to the previous experiment, both monovalently-binding EGFRxCD33 and HER2xCD33 T-BsAbs offered no benefit in controlling tumor growth compared to no treatment ‘tumor only’ and CD33xCD33 control antibody treatment groups.

### EGFR or HER2 single-positive tumors resist EGFRxHER2 T-BsAb treatment

Results from heterodimeric T-BsAbs bearing a Fab with an irrelevant specificity (CD33) indicated that tumor bivalency was required for meaningful tumor control in vivo. We next explored the significance of tumor-binding valency on the specificity and efficacy of the EGFRxHER2 T-BsAb. A CRISPR-Cas9 system was used to knock out either *EGFR* or *HER2* genes in SW1990 cells, generating EGFR^+^ or HER2^+^ single-positive SW1990 lines. The EGFRxHER2 T-BsAb, bearing only one Fab for EGFR and HER2, could be considered ‘tumor monovalent’ in the context of these single-positive PDAC lines.

**Fig. 6:**
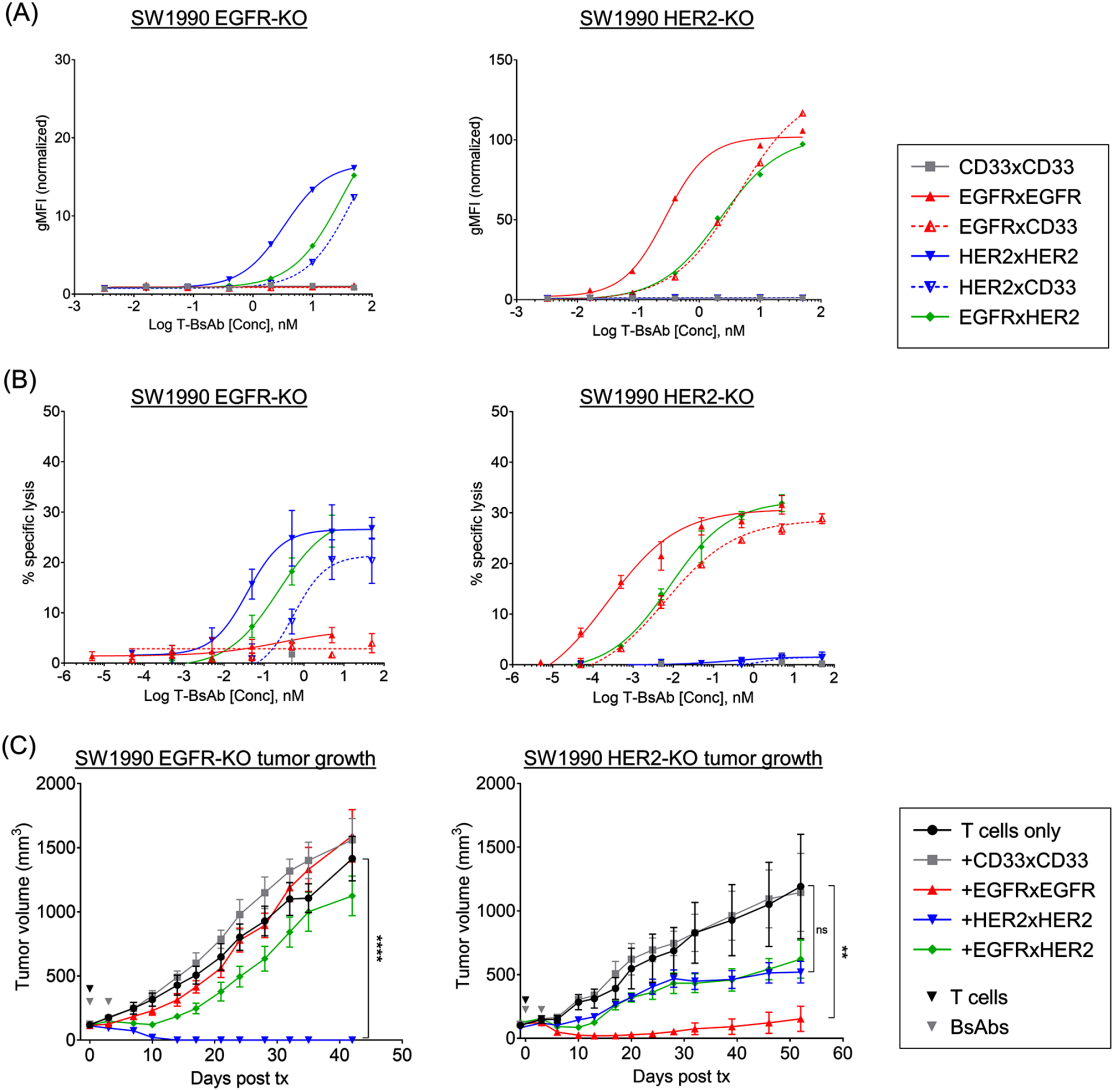
EGFRxHER2 T-BsAbs efficacy is lost in EGFR and HER2-single-positive PDAC (**A**) Mean fluorescent intensities determined by flow cytometry and (**B**) antibody-dependent T cell-mediated cytotoxicity (ADTC) against SW1990-EGFR-KO (left) and SW1990-HER2-KO (right) cell lines. Ratio of effector T cells to target PDAC cells (E: T ratio) was set to 10:1. (**C**) Tumor growth of subcutaneous SW1990 EGFR-KO (left) and HER2-KO (right) tumors. One infusion of 2 × 10^7^ T cells was administered. Two doses of T-BsAbs (10 µg each) were given by i.v. injection

FACS analysis of the SW1990 EGFR-KO and HER2-KO lines revealed reductions in binding strength between the homodimeric T-BsAbs and the EGFRxHER2 T-BsAb as determined by EC50 of the MFIs (Fig. 6A; Table 4). When examining the SW1990 EGFR-KO line, the binding strength of the EGFRxHER2 (EC50 = 31.5nM) and the HER2xCD33 (EC50 = 46.9nM) T-BsAbs were similar, while that of homodimeric HER2xHER2 T-BsAb was ˜ 10-fold stronger (MFI = 3.4nM). Similarly, when applied to the SW1990 HER2-KO line, the EGFRxHER2 and EGFRxCD33 T-BsAbs were comparable in binding strength (EC50 = 2.2nM and 4.4nM respectively), which was ˜ 10 fold worse than the homodimeric EGFRxEGFR (EC50 = 0.3nM).

The EGFRxHER2 T-BsAb mediated cytotoxicity against EGFR-KO and HER2-KO lines with sensitivities comparable to the ‘tumor monovalent’ T-BsAbs HER2xCD33 and EGFRxCD33 respectively (Fig. 6B; Table 4). However, all three heterodimeric T-BsAbs were less effective in driving cytotoxicity against the single-positive targets when compared to the respective homodimeric T-BsAbs.

As expected in both FACS and ADTC analyses, EGFRxEGFR T-BsAbs did not show substantial binding or cytotoxicity on SW1990 EGFR-KO lines, nor did HER2xHER2 T-BsAbs on SW1990 HER2-KO lines (Fig. 6A and B).

In vivo, only HER2xHER2 BsAbs eliminated SW1990 EGFR-KO tumors, as both EGFRxEGFR and EGFRxHER2 T-BsAb-treated tumors grew at rates comparable to groups receiving only T cells or the control CD33xCD33 T-BsAb (Fig. 6C). Conversely, in HER2-KO tumors, EGFRxEGFR BsAbs demonstrated strong tumor control, while HER2xHER2 and EGFRxHER2 T-BsAb treatments had no significant benefit. Both knockout lines appeared to be less aggressive than parental lines, and grew significantly slower than the parental SW1990—KO tumors required ˜ 40 days to grow > 2cm^3^; parental tumors required ˜ 20 days (Figs. 3B and 6C). The reduced aggressiveness of KO tumors coincided with greater sensitivity to T-BsAb treatments, requiring only one dose of T cells to achieve significant antitumor effects. These results highlight the requirement for co-expression of EGFR and HER2 to elicit significant EGFRxHER2 T-BsAb-mediated tumor suppression.

## Discussion

The overwhelming majority of patients with PDAC succumb to the disease, and the frequent expression of EGFR and HER2 in tumors further diminish patient chance of survival. In this manuscript, we reported the generation of EGFR- and HER2-targeting T-BsAbs, as well as a heterodimeric EGFRxHER2 T-BsAb, capable of driving T cell infiltration and eliciting impressive in vivo anti-tumor potency against established cell-derived and patient-derived PDAC xenografts.

FACS analysis of PDAC tumors harvested at an early timepoint in treatment—day 10—when differences between treatments on tumor growth were not yet significant, revealed EGFRxEGFR, HER2xHER2, and EGFRxHER2 T-BsAb treatments increased the presence of TILs, which exhibited a highly activated phenotype denoted by high expression of effector cytokines. Interestingly, in longer time-course treatment experiments, HER2xHER2 and EGFRxHER2 T-BsAbs were more effective at treating PDAC xenografts than EGFRxEGFR T-BsAbs. However, even 10 days after the start of treatment, mice treated with HER2xHER2 and EGFRxHER2 T-BsAbs possessed nearly double the frequency of intratumoral CD11b^+^ host myeloid cells than EGFR homodimer-treated tumors. The increased frequency of a myeloid-rich stroma may be due to HER2xHER2 and EGFRxHER2 T-BsAbs effectively driving the elimination of EGFR^+^HER2^+^ tumor cells while sparing EGFR^−^HER2^−^ stromal cells. Host murine myeloid cells are capable of suppressing anti-tumor functions of transferred human T cells in vivo [37]. Although EGFRxEGFR T-BsAb-treated tumors possessed a lower overall proportion of CD11b^+^ myeloid cells, a significantly higher proportion of these stromal cells expressed PDL1, along with an increased intensity (MFI) of PDL1 expression, consistent with a more immunosuppressive phenotype and a potential roadblock for EGFR T-BsAb treatment.

We have previously shown that depleting tumor-infiltrating macrophages including PMN-MDSCs, M-MDSCs, and tumor associated macrophages significantly improved T-BsAb-mediated T cell infiltration and persistence in tumors, resulting in enhanced anti-tumor immune responses against solid cancers [37]. We demonstrate here that the combinations of EGFRxEGFR T-BsAb treatment with granulocyte and myeloid-depleting therapies (anti-Gr-1 and anti-CSF-1R) drove T cell infiltration into PDAC tumors and improved anti-tumor efficacy without side effects.

Homodimeric EGFR T-BsAbs consistently demonstrated greater cell surface binding and cytotoxicity to PDAC cell lines compared homodimeric HER2 T-BsAbs. Although EGFR and HER2 were coexpressed on nearly every PDAC cell line and patient-derived PDAC xenograft that we reported, these cells and tissues appeared to consistently express higher levels of EGFR compared to HER2. In vitro, the higher expression of EGFR likely explains to greater sensitivity to EGFRxEGFR T-BsAbs. This is consistent with FACS and ADTC assays on the OSCA-40 canine osteosarcoma line which expressed low but comparable levels of EGFR and HER2 immunoreactivity (Supp. Figure 5, Supp. Table 2). Homodimeric EGFR and HER2 T-BsAbs exhibited comparable cell surface binding, with the HER2 T-BsAbs achieving a lower ADTC EC50 than EGFR T-BsAbs against OSCA-40 cells.

In vivo, however, the homodimeric HER2 T-BsAbs achieved dramatically superior therapeutic benefits, whereas the EGFRxEGFR T-BsAb only conferred modest delays in tumor growth. The exceptional in vivo potency of the HER2xHER2 and EGFRxHER2 T-BsAbs compared to the EGFRxEGFR T-BsAb may be attributed at least in part to the substantially higher affinity of the HER2-specific Fab compared to the EGFR Fab (K_D_: EGFRxEGFR = 1.33E-11 M; HER2xHER2 = 1.12E-13 M). The pancreatic tumor microenvironment tends to have a fibrotic, desmoplastic, poorly vascularized, and stroma-rich setting that may negatively impact intratumoral T-BsAb circulation, binding, and T cell activation and infiltration. The superior avidity of the HER2 T-BsAb may contribute to improved persistence of tumor cell surface binding and consequently facilitate enhanced T cell engagement.

Discrepancy between in vitro cytotoxicity and in vivo tumor efficacy has been observed repeatedly in this T-BsAb tumor model [19, 22, 23], where strong in vitro activity did not predict the in vivo effect. Despite discrepant results between EGFR and HER2 homodimeric T-BsAbs in vitro and in vivo, the efficacy of the EGFRxHER2 heterodimer closely matched the efficacy of the more effective homodimeric T-BsAb in either context. FACS, IHC, and ADTC analyses revealed the EGFRxHER2 T-BsAb to be nearly as effective as the superior homodimeric EGFR T-BsAb. It is again worth noting that all parental PDAC lines expressed higher levels of EGFR than HER2. However, OSCA-40 cells, which express low and comparable levels of EGFR and HER, exhibited greater sensitivities to HER2xHER2 and EGFRxHER2 T-BsAbs than to the EGFRxEGFR homodimer (Supp. Figure 5, Supp. Table 2). The HER2 homodimer and EGFRxHER2 heterodimer achieved similar EC50s. These results suggest tissues and tumors with low expression of both EGFR and HER2 are not any more or less sensitive to heterodimeric T-BsAbs than to homodimers. In vivo, the EGFRxHER2 T-BsAb achieved comparable survival benefits relative to the HER2xHER2 T-BsAb. In these contexts, tumor bivalency appeared to be required for the EGFRxHER2 T-BsAb to achieve results comparable to the homodimeric T-BsAb in a given experimental setting. For example, even though HER2 T-BsAbs were more effective in treating SW1990 tumors than EGFR T-BsAbs, pairing the HER2 Fab with the less effective EGFR Fab in the heterodimeric EGFRxHER2 BsAb did not significantly diminish the overall anti-tumor efficacy. Conversely, ‘tumor monovalent’ T-BsAbs demonstrated dramatically impaired anti-tumor responses. EGFRxCD33 and HER2xCD33 T-BsAbs exerted no control over EGFR^+^HER2^+^ cell-derived and patient-derived PDAC xenografts. EGFRxHER2 T-BsAbs are also effectively ‘tumor monovalent’ when applied to EGFR^+^ or HER2^+^ single-positive tumors. Consequently, treatment of SW1990 EGFR-KO or HER2-KO tumors with the ‘tumor monovalent’ EGFRxHER2 T-BsAb failed to confer any therapeutic benefit. The high specificity of the heterodimeric EGFRxHER2 T-BsAb for EGFR^+^HER2^+^ double-positive PDAC tumors could offer a strategy to spare healthy single-positive tissues and reduce on-target/off-tumor side effects.

Efforts to conditionally target EGFR and HER2 co-expression have been explored using colocalization-dependent protein switches (Co-LOCKR) engineered into CAR T cells [42]. Tuning of EGFR and HER2 binding moieties allowed for optimization in activation and cytotoxic responses against transgenic EGFR^+^ and HER2^+^ cell lines. The EGFRxHER2 T-BsAbs described here utilize EGFR- and HER2-specific Fabs based on amino acid sequences of clinically approved antibodies cetuximab and trastuzumab. Fine-tuning the binding affinities of tumor-targeting Fabs, with consideration for tumor antigen expression levels, may similarly improve EGFRxHER2 efficacy and tumor specificity.

Our analysis of 22 patient-derived PDAC xenografts and 7 established human PDAC cell lines revealed frequent co-expression of EGFR and HER2 in PDAC (22.7% of PDXs co-expressed EGFR and HER2 detected by IHC, all seven PDAC cell lines exhibited co-expression measured by flow cytometry) (Supp. Table 1, Fig. 1B). This is consistent with another study that found frequent co-expression of EGFR and HER2 in PDAC samples [43]. However, tumor tissues are not exclusive in their co-expression of EGFR and HER2. According data found in the Human Protein Atlas, normal tissues that share ‘medium’ or higher expression of both EGFR and HER2 include skin, nasopharynx (‘high’ expression of EGFR), testis, and some female reproductive tissues (placenta, cervix, fallopian tubes) [44, 45]. EGFRxHER2 T-BsAbs demonstrated unique specificity for EGFR^+^HER2^+^ double-positive tumors. Although the toxicities of the clinically-approved EGFR- and HER2-targeting moieties used in EGFRxHER2 T-BsAbs (cetuximab and trastuzumab respectively) do not typically overlap and are generally well-tolerated, the potential on-target/off-tumor side effects of EGFRxHER2 T-BsAbs will need to be carefully monitored.

## Conclusions

In this study we developed EGFR- and HER2-directed T-BsAbs that demonstrated impressive antitumor efficacy in PDAC xenografts. We further developed heterodimerized EGFRxHER2 T-BsAbs possessing one EGFR-specific Fab and one specific for HER2. EGFRxHER2 T-BsAbs demonstrated antitumor efficacy in PDAC xenografts comparable to homodimeric EGFR and HER2 T-BsAbs. However, heterodimerized T-BsAbs required both tumor-binding moieties for effective antitumor effectiveness, as loss of either EGFR or HER2 Fabs failed to exhibit a significant benefit in tumor control. Similarly, loss of either EGFR or HER2 expression in PDACs xenografts abrogated the therapeutic effect of EGFRxHER2 T-BsAbs in vivo. These results contrast with homodimeric EGFR or HER2 T-BsAbs (bearing two Fabs for either EGFR or HER2) that maintained efficacy against EGFR^+^ or HER2^+^ single-positive xenografts respectively. The described heterodimeric T-BsAb platform has the potential to leverage differences in avidity between double-positive tumors and other tissues that either express one or weakly express both targets to improve specificity to tumors. Heterodimerization of T-BsAbs has the potential to reduce on-target/off-tumor complications by limiting effects to double-positive tumor tissues.

### Electronic supplementary material

Below is the link to the electronic supplementary material.

**Supplementary Material 1:** Supplementary Figures and Tables

## Data Availability

All data needed to evaluate the conclusions in the article are present in the article and/or the Supplementary Materials. The data and materials used in the current study are available from the corresponding authors upon reasonable request.

## Abbreviations

T-BsAb: T cell-engaging bispecific antibody
PDAC: pancreatic ductal adenocarcinoma
IHC: immunohistochemistry
CDX: cell-derived xenograft
PDX: patient-derived xenograft
