# Supplementary material for: Heterodimerization of T cell engaging bispecific antibodies to enhance specificity against pancreatic ductal adenocarcinoma
Source: J Hematol Oncol. 2024 Apr 23;17:20. doi: 10.1186/s13045-024-01538-5 (PMC11036555; doi:10.1186/s13045-024-01538-5)
Supplement: Supplementary file 1 — Supplementary Material 1: Supplementary Figures and Tables [file 13045_2024_1538_MOESM1_ESM.docx]

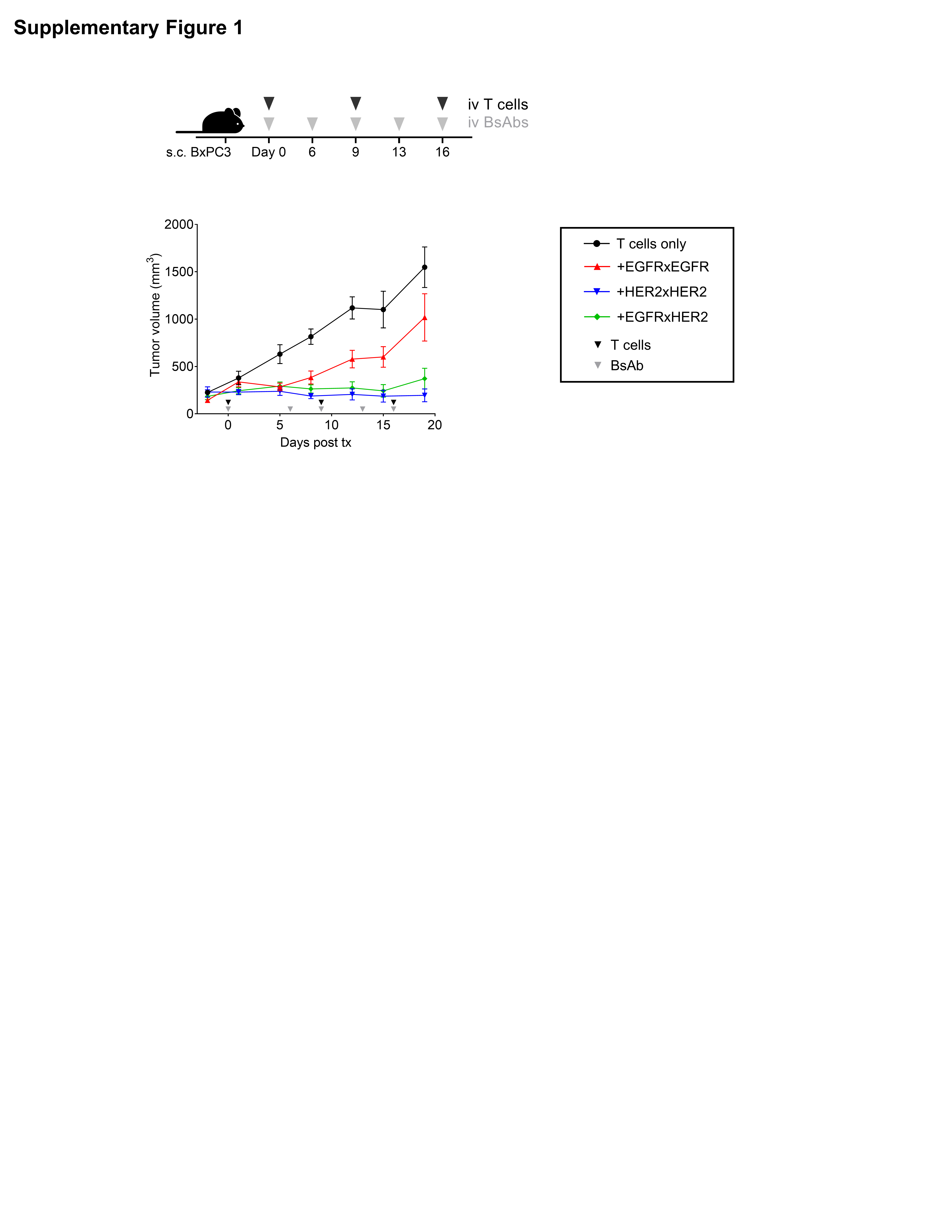
**Supplementary Figure 1. Heterodimeric EGFR and HER2 T-BsAbs impede BxPC-3 growth**

*In vivo* antitumor effect of T-BsAbs in the presence of human T cells against BxPC-3 cell line xenografts. 3×10^6^ BxPC-3 cells were subcutaneously implanted into mice (n=4 mice per group). 10µg T-BsAbs were administered twice per week and 2×10^7^ T cells were administered once per week for 2 weeks to treat the tumors.


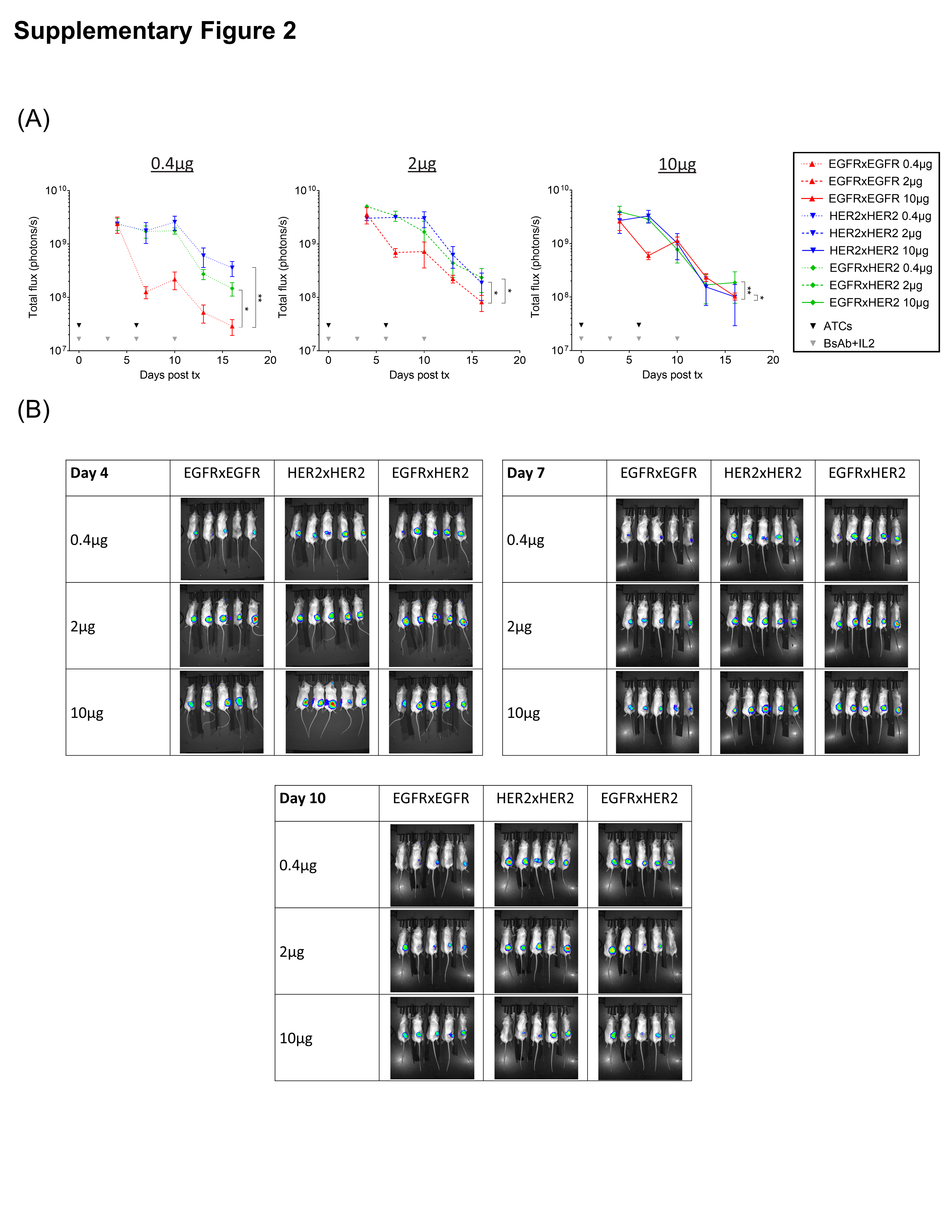
**Supplementary Figure 2. T cell tumor homing responses to EGFR and HER2 T-BsAbs**

(A) Bioluminescence imaging (BLI) of Luc(+) T cells over time. Only the first infusion of T cells were Luc(+) T cells. Quantitation of bioluminescence intensity in the lesions of tumors. Mice were treated with 0.4µg, 2µg, and 10µg doses of T-BsAbs. Differences between curves were determined by one-way ANOVA with Tukey’s post hoc test on log-transformed values of the area under the curves. (B) Representative images were taken on day 4, day 7, and day 10.

**
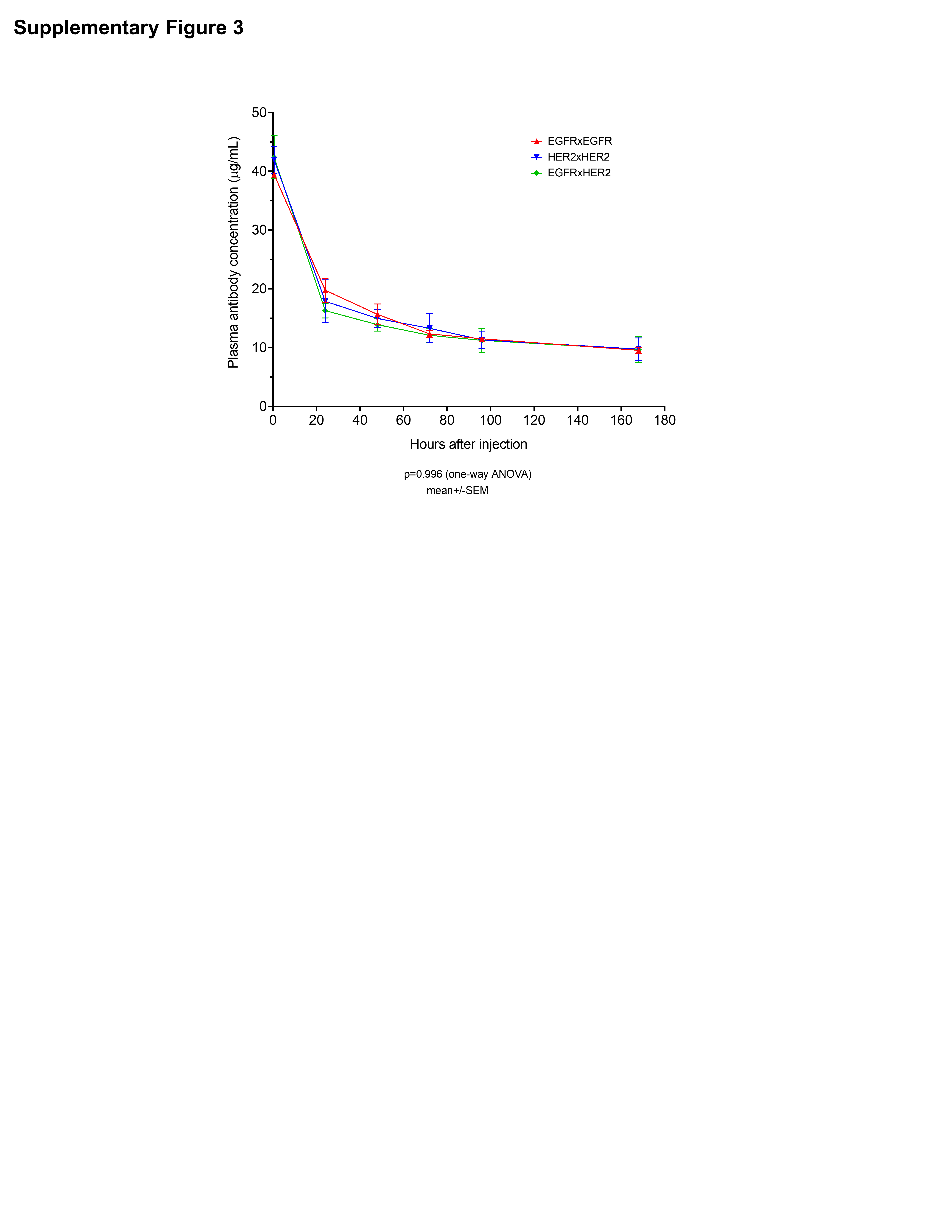
Supplementary Figure 3. T-BsAbs share similar pharmacokinetics**

Plasma concentrations of EGFR and HER2 T-BsAbs in tumor-free mice over time. Mice (n=5) were administered a single dose of T-BsAbs (100µg). Mice were serially bled over 7 days (0.5hr – 168hrs) and concentrations were determined by ELISA. Area under the curve (AUC) for each T-BsAb is provided.


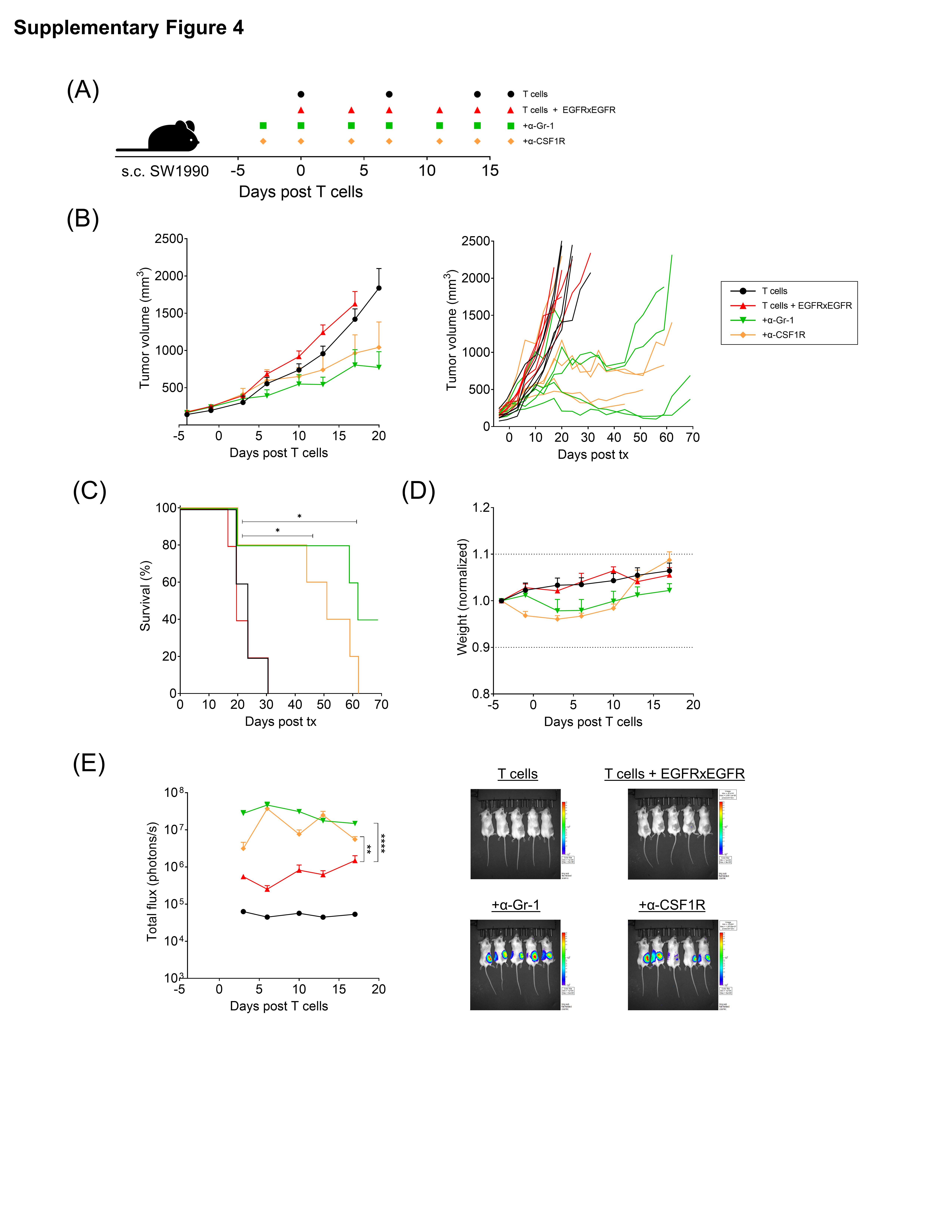
**Supplementary Figure 4. Myeloid cell depletion improves overall survival in PDAC-bearing mice and enhances EGFRxEGFR T-BsAb-mediated T cell tumor infiltration**

*In vivo* effect of EGFRxEGFR T-BsAbs in combination with myeloid cell depletion. (A) SW1990 tumor-bearing mice were pre-treated with 100µg of anti-mouse GR-1 or 100µg of anti-mouse CSF-1R to deplete myeloid cells 3 days prior to treatment. Starting on day 0, 10µg EGFRxEGFR T-BsAbs were administered twice per week and 2×10^7^ T cells were administered once per week for 2 weeks. (B) Tumor growth displayed as combined (left) or individual (right) growth curves. (C) Overall survival and (D) relative body weight of mice during treatment were plotted. (E) Bioluminescence imaging (BLI) of Luc(+) T cells. Quantitation of bioluminescence intensity in the lesions of tumors. Representative images (right) were taken on day 6 post-treatment initiation.

**
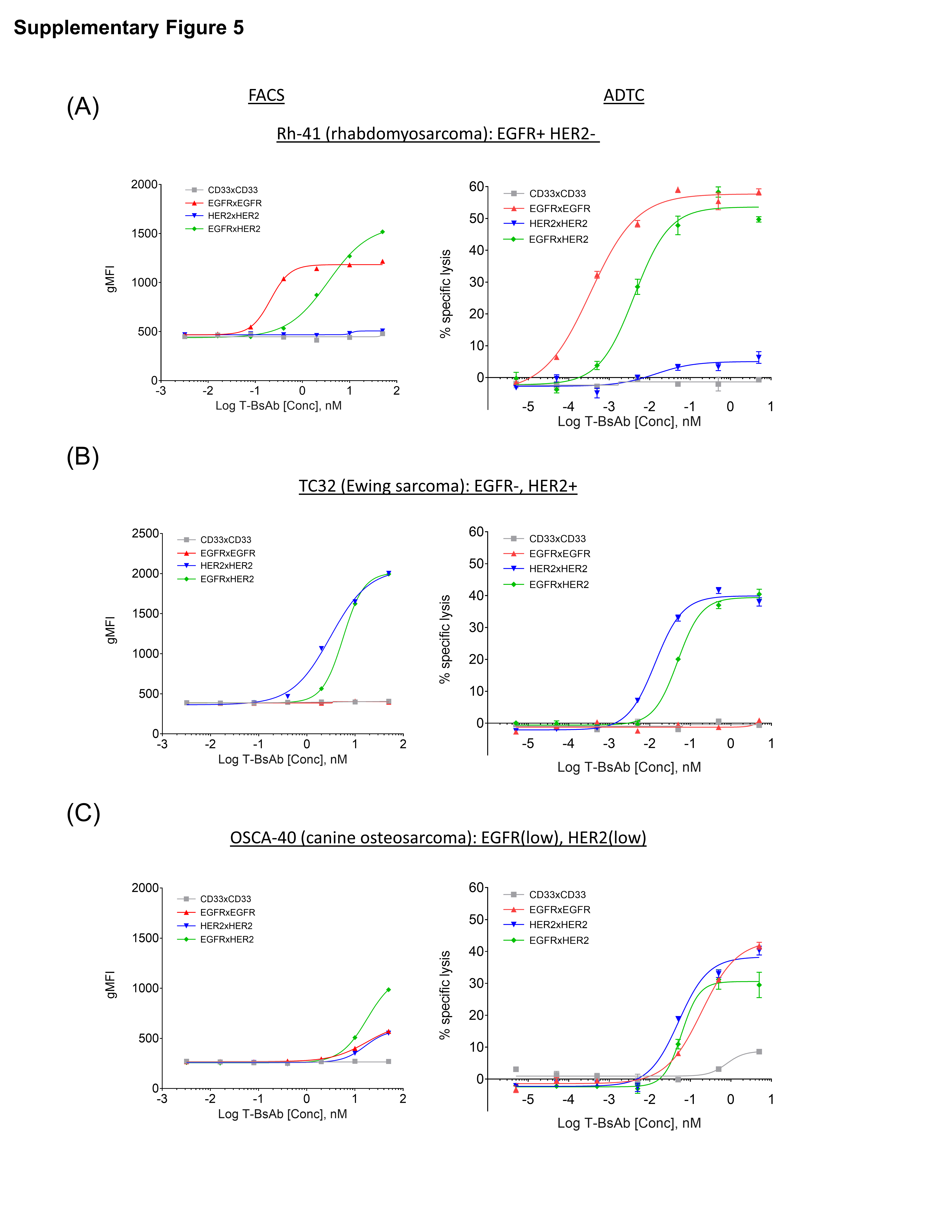
Supplementary Figure 5. EGFRxHER2 T-BsAbs exhibit diminished binding and cytotoxicity to EGFR or HER2 single-positive tumor cells compared to respective EGFR or HER2 homodimeric T-BsAbs**

Mean fluorescent intensities determined by flow cytometry (left) and antibody-dependent T cell-mediated cytotoxicity (ADTC) (right) against (A) Rh-41 rhabdomyosarcoma (EGFR+ HER2-), (B) TC32 Ewing sarcoma (EGFR-, HER2+), and (C) OSCA-40 canine osteosarcoma (EGFR(low), HER2(low)). Ratio of effector T cells to target PDAC cells (E:T ratio) was set to 10:1.

**Supplementary Table 1 Summary of pancreatic adenocarcinoma patient-derived xenografts**

| **PDX #** | **AJCC Staging at time of collection** | **Differentiation** | **Primary or Metastasis (Met)** | **EGFR expression (1-3)** | **HER2 expression (1-3)** |
| --- | --- | --- | --- | --- | --- |
| 1 | Stage IIB T3N1M0 | Moderately differentiated | Primary | 1 | 0 |
| 2 | Stage IIB T3N1M0 | Moderately differentiated | Primary | 1 | 1 |
| 3 | Stage III T2N2M0 | Moderately differentiated | Primary | 0 | 0 |
| 4 | Stage Ib T2N0M0 | Moderately differentiated | Primary | 1 | 1 |
| 5 | Stage IV T4N2M1 | Unavailable | Met | 2 | 0 |
| 6 | Stage IV T4N2M1 | Unavailable | Met | 2 | 2 |
| 7 | Stage IV T4N2M1 | Moderately differentiated | Primary | 3 | 0 |
| 8 | Stage IV TXN2M1 | Moderately differentiated | Primary | 1 | 0 |
| 9 | Stage IIB T3N1M0 | Moderately differentiated | Primary | 1 | 0 |
| 10 | Stage IV T4N0M1 | Moderately differentiated | Met | 2 | 1 |
| 11 | Stage IIB T3N1M0 | Moderately differentiated | Primary | 0 | 3 |
| 12 | Stage IIA T3N | Moderately differentiated | Primary | 0 | 0 |
| 13 | Stage IIB T3N1M0 | Well differentiated | Primary | 1 | 0 |
| 14 | Stage IIB T3N1M0 | Moderate/Poor differentiated | Primary | 1 | 0 |
| 15 | Stage IIB T3N1M0 | Moderately differentiated | Primary | 1 | 0 |
| 16 | Stage Ib T2N0M0 | Moderately differentiated | Primary | 1 | 0 |
| 17 | Stage IIB T3N1M0 | Moderate/Poor differentiated | Primary | 2 | 1 |
| 18 | Stage IV T4N0M1 | Unavailable | Met | 1 | 0 |
| 19 | Stage IV T2N0M1 | Moderately differentiated | Met | 0 | 0 |
| 20 | Stage IV T2N0M1 | Moderately differentiated | Met | 0 | 0 |
| 21 | Stage T3N0M1 | Unavailable | Met | 0 | 0 |
| 22 | Stage IIIC T2N2M0 | Moderately differentiated | Primary | 0 | 0 |

| **Supplementary Table 2 In vitro binding (FACS) and cytotoxic sensitivities (ADTC) to bispecific antibodies in tumor cell lines with variable expression of EGFR and HER2** | | | | | |
| --- | --- | --- | --- | --- | --- |
| **Cell line** | **CD33xCD33** | | **EGFRxEGFR** | **HER2xHER2** | **EGFRxHER2** |
| **FACS binding (EC50, pM)** | | | |  |  |
| Rh-41 (EGFR+ HER2-) | — | 211 | | — | 3546 |
| TC32 (EGFR-, HER2+) | — | — | | 3075 | 5652 |
| OSCA-40 (EGFR-low, HER2-low) | — | 17440 | | 16330 | 17890 |
|  |  |  | |  |  |
| **ADTC (EC50, pM)** | | | |  |  |
| Rh-41 (EGFR+ HER2-) | — | 0.3 | | ~14.2 | 4.2 |
| TC32 (EGFR-, HER2+) | — | — | | 13.6 | 48.8 |
| OSCA-40 (EGFR-low, HER2-low) | — | 188.6 | | 51.4 | 60.4 |
| Rh-41 rhabdomyosarcoma; TC32 Ewing sarcoma; OSCA-40 canine osteosarcoma. | | | | | |
